# Supplementary material for: Can Disruption of Basal Ganglia-Thalamocortical Circuit in Wilson Disease Be Associated with Juvenile Myoclonic Epilepsy Phenotype?
Source: Brain Sci. 2022 Apr 26;12(5):553. doi: 10.3390/brainsci12050553 (PMC9138607; doi:10.3390/brainsci12050553)
Supplement: Supplementary file 1 [file brainsci-12-00553-s001.zip › Supplementary Tables.pdf]

Supplementary Table S1: Regions of increased and decreased functional connectivity of left M1
Cluster-size threshold k= 87 voxels, corrected at α < 0.05.

	Side	Cluster	Voxel level	MNI coordinates			Talairach coordinates		
**Brain areas**		**k**	**Z**	**x**	**y**	**z**	**x**	**y**	**z**
Decreased connectivity									
Parahippocampal gyrus (BA 37)	l	93	5.23	-26	-46	-14	-26	-45	-10
			4.86	-30	-54	-12	-30	-53	-7
			3.82	-24	-60	-20	-24	-59	-14
Cuneus and lingual gyrus (BA 17)	l	100	4.76	-16	-74	8	-16	-71	11
			4.2	-22	-68	2	-22	-66	5
			3.65	-4	-78	8	-4	-75	11
Increased connectivity									
Post-central gyrus, inferior parietal lobule, supramarginal gyrus (BA 5 and BA 40)	r	241	7.06	32	-42	54	32	-38	52
			6.15	30	-44	68	30	-39	65
			4.73	24	-44	58	24	-40	55
Post-central gyrus, supramarginal gyrus (BA 2 and BA 40)	r	163	6.63	44	-40	52	44	-36	50
			4.8	50	-30	42	50	-27	40
Post-central gyrus, inferior parietal lobule (BA 2 and BA 40)	l	396	6.07	-42	-38	52	-42	-34	50
			5.64	-46	-26	32	-46	-24	31
			4.98	-38	-36	40	-38	-33	39
Pre-central gyrus (BA 6)	l	89	5.76	-24	-18	56	-24	-15	52

Supplementary Table S2: Regions of increased functional connectivity of right M1
Cluster-size threshold k= 84 voxels, corrected at α < 0.05.

	**Side**	**Cluster**	**Voxel level**	**MNI coordinates**			**Talairach coordinates**		
**Brain areas**		**k**	**Z**	**x**	**y**	**z**	**x**	**y**	**z**
Increased connectivity									
Supramarginal and post-central gyrus (BA 40)	r	124	7.53	32	-42	54	32	-38	52
			4.52	28	-40	44	28	-37	42
Post-central gyrus, inferior parietal lobule (BA and BA 2)	r	117	6.69	54	-28	44	53	-25	42
			5.64	44	-40	54	44	-36	52
Post-central gyrus (BA 3)	r	109	6.04	44	-26	54	44	-23	51
			4.65	34	-28	64	34	-24	60

Supplementary Table S3: Regions of increased and decreased functional connectivity of left GP
Cluster-size threshold k= 95 voxels, corrected at α < 0.05.

	Side	Cluster	Voxel level	MNI coordinates			Talairach coordinates		
**Brain areas**		**k**	**Z**	**x**	**y**	**z**	**x**	**y**	**z**
Decreased connectivity									
Cerebellum, brainstem	l/r	302	7.19	14	-32	-22	14	-32	-17
			6.96	-8	-30	-20	-8	-30	-15
			6.32	-14	-26	-16	-14	-26	-12
Body of caudate nucleus	l/r	431	6.43	-2	4	16	-2	5	15
			5.88	-6	18	10	-6	18	8
			5.36	-10	8	20	-10	9	18
Increased connectivity									
Thalamus (anterior nuclei), head of caudate nucleus	r/l	420	7.72	8	-8	14	8	-7	13
			7.09	-6	10	16	-6	-9	15
			6.93	6	4	0	6	4	0
Post-central gyrus (medial part), mid cingulate (BA 5 and BA 31)	l/r	199	6.28	4	-36	54	4	-32	51
Post and pre-central gyrus (BA 3 and BA 4)	l	110	5.37	-42	-22	42	-42	-19	40
			4.32	-36	-18	52	-36	-15	49
Post and pre-central gyrus (BA 3, BA 4 and BA 6)	r	279	4.97	8	-36	68	8	-32	64
			4.54	14	-32	64	14	-28	60
			4.51	28	-30	60	28	-26	57
Post and pre-central gyrus (BA 3 and BA 4)	l	260	4.82	-8	-40	68	-8	-36	64
			4.76	-24	-30	52	-24	-27	49
			4.68	-38	-28	56	-38	-25	53

Supplementary Table S4: Regions of increased and decreased functional connectivity of right GP
Cluster-size threshold k= 108 voxels, corrected at α < 0.05.

	Side	Cluster	Voxel level	MNI coordinates			Talairach coordinates		
**Brain areas**		**k**	**Z**	**x**	**y**	**z**	**x**	**y**	**z**
Decreased connectivity									
Cerebellum, brainstem	l/r	447	>8	12	-30	-17	12	-30	-17
			>8	10	-40	-18	10	-40	-13
			>8	-8	-30	-20	-8	-30	-15
Body of the caudate nucleus	l/r	482	>8	-4	-2	18	-4	-1	17
			7.49	8	-2	20	8	-1	18
			6.11	-6	18	10	-6	18	8
Cuneus, lingual gyrus, parahippocampal gyrus, posterior Cingulate (BA 17, BA 18, BA 28, BA 23, BA 30)	l/r	1002	7.28	24	-68	2	24	-66	5
			6.56	-8	-80	10	-8	-77	13
			6.05	26	-52	0	26	-50	3
Precuneus, middle occipital gyrus (BA 7 and BA 19)	r	220	6.08	30	-72	38	30	-68	38
			5.53	30	-84	24	30	-80	26
			4.45	32	-76	24	32	-73	26
Precuneus, middle occipital gyrus (BA 7 and BA 19)	l	236	5.89	-30	-72	42	-30	-68	42
			5.69	-26	-76	34	-26	-72	35
			5.14	-28	-86	18	-28	-82	21
Cerebellum, fusiform gyrus (BA 37)	r	114	4.67	28	-58	-22	28	-57	-16
			4.34	28	-52	-16	28	-51	11
			4.16	32	-36	-20	32	-36	-15
Increased connectivity									
Head and bod of caudate nucleus, Thalamus (anterior nuclei)	l/r	682	>8	6	4	0	6	4	0
			>8	6	-8	14	6	-7	13
			>8	-4	0	8	-4	0	7
Thalamus (pulvinar)	l/r	111	6.88	-18	-30	14	18	-28	14
			5.78	6	-30	6	6	-29	7
			4.77						
Pre and post-central gyrus (BA 3 and BA 4)	l	318	6.08	-24	-30	52	-24	-27	49
			4.76	-20	-40	56	-20	-36	53
			4.56	-10	-34	68	-10	-30	64
Pre and post-central gyrus (BA 3 and BA 4)	r	364	5.81	42	-24	50	42	-21	47
			5.3	14	-34	62	14	-30	59
			5.15	28	-32	62	28	-28	59
Post-central gyrus (BA 3 and BA 2)	l	109	5.26	-42	-22	42	-42	-19	40
			5.07	-36	-24	36	-36	-22	34
			4.27	-48	-18	46	-48	-15	43
