# Supplementary material for: Can Disruption of Basal Ganglia-Thalamocortical Circuit in Wilson Disease Be Associated with Juvenile Myoclonic Epilepsy Phenotype?
Source: Brain Sci. 2022 Apr 26;12(5):553. doi: 10.3390/brainsci12050553 (PMC9138607; doi:10.3390/brainsci12050553)
Supplement: Supplementary file 1 [file brainsci-12-00553-s001.zip › Supplementary text.pdf]

Multimodal imaging study

Voxel based morphometry (VBM)

Image acquisition. A high-resolution T1-weighted anatomical image was acquired for the patient (170 sagittal slices; TR= 9.9 ms; TE= 4.6 ms; in plane matrix= 256x256; voxel size=1x1x1 mm) and for each healthy participant (360 sagittal slices; TR= 9.9 ms; TE= 4.6 ms; in plane matrix= 480x480; voxel size=0.5x0.5x0.5 mm).

Data preprocessing and analysis. Data were preprocessed and analyzed according to the optimized method:[1] images were first spatially normalized to the same stereotaxic space by registering each image to the same template, segmented into different tissue classes; images were adjusted incorporating the relative voxel volumes thus allowing to compare the absolute amount of grey matter in different regions.

Finally, a statistical analysis using the general linear model (GLM) was used to identify regions of grey matter volume that are significantly different in patient with respect to controls. Total intracranial volume was used as covariate of no interest.

Resting-state fMRI

Protocol. During resting-state sessions, patient and controls were instructed to keep eyes closed and to relax without falling asleep. Gradient-echo echo-planar sequences were acquired on a Philips Achieva system at 3T with the following parameters: thirty-five axial contiguous slices (TR=2050 ms or 2000 ms; in-plane matrix=80x80; voxel size: 3.6x3.6x3.6; 200 volumes) for healthy subject, and thirty slices dataset (TR=2000 ms; in-plane matrix=80x80; voxel size: 3x3x4; 240 volumes) for the patient.

Data pre-processing. MATLAB version R2013a (The MathWorks Inc, Natick, Mass) and SPM12 (Wellcome Department of Imaging Neuroscience, London, UK) were used for functional data pre-processing and analysis. Patient and healthy controls functional volumes were pre-processed applying the following procedure: slice timing correction, realignment to the first volume acquired,

normalization to the MNI (Montreal Neurologic Institute) template implemented in SPM12, temporal filtering (0.008-0.1 Hz) using Resting-State fMRI Data Analysis Toolkit (REST),[2] and spatial smoothing using a 4 mm FWHM Gaussian kernel.

During scanning, scalp EEG has been recorded by means of a 32-channel MRI-compatible EEG recording system (Micromed, Mogliano Veneto, Italy). Electrodes were placed according to conventional 10–20 locations.

Seed-based functional connectivity analyses. To evaluate the correlation between the signal of selected regions of interest (ROIs) and each voxel within the brain seed-based functional connectivity analyses were run using several seeds. Left and right M1 and globus pallidus of the AAL atlas were used as ROIs (Tzourio-Mazoyer et al., 2002),[3] whereas the Supplementary Motor Area (SMA) was defined as a 10-mm radius sphere with peak coordinates in MNI space, $x = -2$, $y = -10$, $z = 59$ (Mayka et al., 2006).[4] The BOLD signal time course within each ROI was extracted by means of marsbar (http://marsbar.sourceforge.net/).

Five separate first level regression analyses were performed for each subject (healthy participants and patient), using the mean signal time course of the seed regions as predictor. The six head-motion parameters (translations and rotations), and mean signal fluctuations in gray matter, white matter and cerebrospinal fluid were used as nuisance regressors.

Patient and healthy controls contrast images were generated for each seed and were then included in second-level two-sample T-tests. A double statistical threshold (voxel-wise $p < 0.001$ and spatial extent) was adopted to achieve a combined significance, corrected for multiple comparisons, of $\alpha < 0.05$, as computed by 3dClustSim AFNI routine, using the "-acf" option (https://afni.nimh.nih.gov/pub/dist/doc/program_help/3dClustSim.html).

For all analyses, the Matthew Brett correction (mni2tal: http://www.mrc-cbu.cam.ac.uk/Imaging/mnispace.html) was applied to the SPM-MNI coordinates to obtain the coordinates in Talairach space.[5]

EEG-fMRI data processing After offline correction of the gradient artifacts and filtering of the EEG signal (Allen et al., 2000), the EEG data were reviewed and pre-processed according to our previous published method (Avanzini et al., 2014, Ruggieri et al., 2015).[6,7] Two experienced electroencephalographers reviewed the pre-processed EEG recordings independently in order to identify spikes. After identification, epileptiform activities were manually marked on EEG and exported as single event or variable-length blocks depending on their duration observed by EEG.

Bibliography

1. Ashburner J.; Friston, K.J. *Human Brain Function,* 2nd ed; Ashburner J FK and PW, 2003.
2. Song, X-W.; Dong, Z-Y.; Long, X-Y.; et al. REST: A Toolkit for Resting-State Functional Magnetic Resonance Imaging Data Processing. *Harrison BJ,* ed. PLoS One **2011**, *6(9).*
3. Tzourio-Mazoyer, N.; Landeau, B.; Papathanassiou, D.; et al. Automated anatomical labeling of activations in SPM using a macroscopic anatomical parcellation of the MNI MRI single-subject brain. *Neuroimage* **2002**, *15(1)*, 273-289.
4. Mayka, M.A.; Corcos, D.M.; Leurgans, S.E.; Vaillancourt, D.E.; Three-dimensional locations and boundaries of motor and premotor cortices as defined by functional brain imaging: A meta-analysis. *Neuroimage* **2006**, *31(4)*, 1453-1474.
5. Talairach, J.; Tour-noux P. *Co-Planar Stereotaxic Atlas of the Human Brain*. Thieme Medical Publisher, 1988.
6. Avanzini, P.; Vaudano, A.E.; Vignoli, A.; et al. Low frequency mu-like activity characterizes cortical rhythms in epilepsy due to ring chromosome 20. *Clin Neurophysiol* **2014**, *125(2),* 239-249.
7. Ruggieri, A.; Vaudano, A.E.; Benuzzi, F.; et al. Mapping (and modeling) physiological movements during EEG-fMRI recordings: The added value of the video acquired simultaneously. *J Neurosci Methods* **2015**, *239*, 223-237.
