# Supplementary figures and images for: Can Disruption of Basal Ganglia-Thalamocortical Circuit in Wilson Disease Be Associated with Juvenile Myoclonic Epilepsy Phenotype?
Source: Brain Sci. 2022 Apr 26;12(5):553. doi: 10.3390/brainsci12050553 (PMC9138607; doi:10.3390/brainsci12050553)

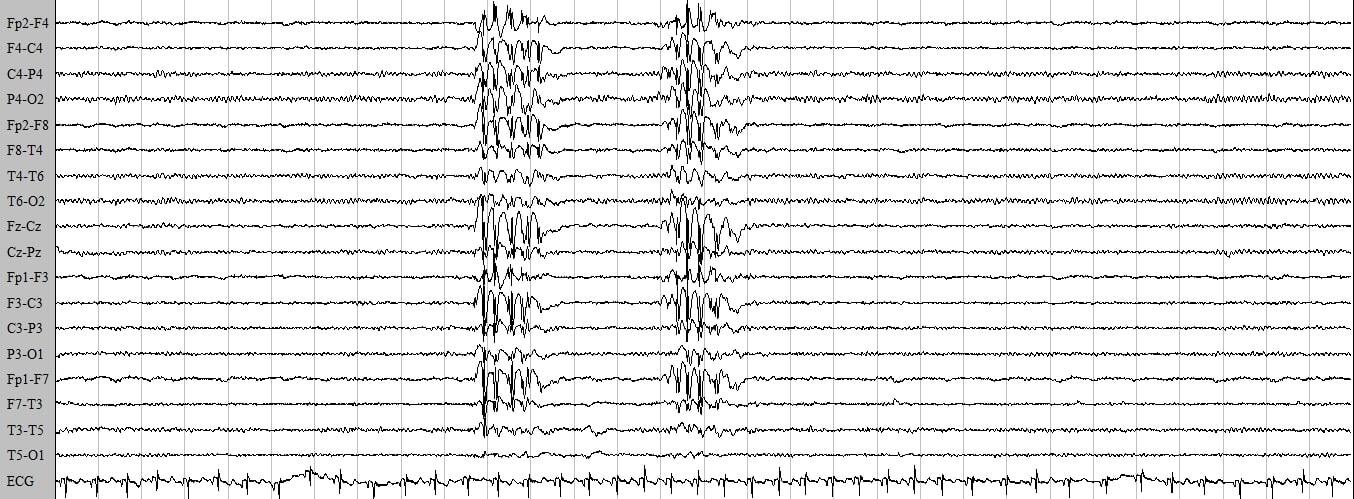

Supplement: Supplementary file 1 [file brainsci-12-00553-s001.zip › Supplementary Figure S1.jpeg]

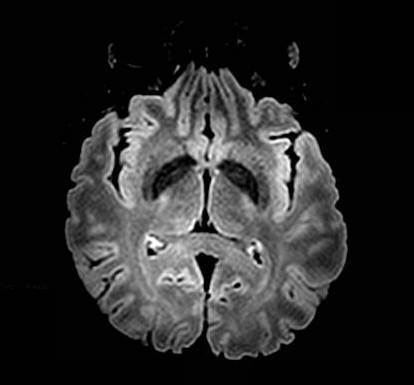

Supplement: Supplementary file 1 [file brainsci-12-00553-s001.zip › Supplementary Figure S2.jpeg]
